# The Beijing genotype and drug resistant tuberculosis in the Aral Sea region of Central Asia

**DOI:** 10.1186/1465-9921-6-134

**Published:** 2005-11-08

**Authors:** Helen Suzanne Cox, Tanja Kubica, Daribay Doshetov, Yared Kebede, Sabine Rüsch-Gerdess, Stefan Niemann

**Affiliations:** 1 Médecins Sans Frontières (MSF), Aral Sea Area Programme, Uzbekistan and Turkmenistan Tashkent, Uzbekistan; 2 National Reference Center for Mycobacteria, Forschungszentrum Borstel, Borstel, Germany; 3 Ministry of Health, Nukus, Karakalpakstan, Uzbekistan; 4 Médecins Sans Frontières (MSF), Amsterdam, Holland

## Abstract

**Background:**

After the collapse of the Soviet Union, dramatically increasing rates of tuberculosis and multidrug-resistant tuberculosis (MDR-TB) have been reported from several countries. This development has been mainly attributed to the widespread breakdown of TB control systems and declining socio-economic status. However, recent studies have raised concern that the Beijing genotype of *Mycobacterium tuberculosis *might be contributing to the epidemic through its widespread presence and potentially enhanced ability to acquire resistance.

**Methods:**

A total of 397 *M. tuberculosis *strains from a cross sectional survey performed in the Aral Sea region in Uzbekistan and Turkmenistan have been analysed by drug susceptibility testing, IS*6110 *fingerprinting, and spoligotyping.

**Results:**

Fifteen isolates showed mixed banding patterns indicating simultaneous infection with 2 strains. Among the remaining 382 strains, 152 (40%) were grouped in 42 clusters with identical fingerprint and spoligotype patterns. Overall, 50% of all isolates were Beijing genotype, with 55% of these strains appearing in clusters compared to 25% of non-Beijing strains. The percentage of Beijing strains increased with increasing drug resistance among both new and previously treated patients; 38% of fully-susceptible isolates were Beijing genotype, while 75% of MDR-TB strains were of the Beijing type.

**Conclusion:**

The Beijing genotype is a major cause of tuberculosis in this region, it is strongly associated with drug resistance, independent of previous tuberculosis treatment and may be strongly contributing to the transmission of MDR-TB. Further investigation around the consequences of Beijing genotype infection for both tuberculosis transmission and outcomes of standard short course chemotherapy are urgently needed.

## Background

Tuberculosis (TB) remains one of the leading infectious killers worldwide, with an estimated 2 million deaths annually (1). In the year 2002 the number of incident TB cases was estimated at 8.8 million (2). Globally there is a 1.8% annual rise in new tuberculosis cases, with a 6% yearly increase in the former Soviet Union (1).

These data are increasingly accompanied by the phenomenon of drug-resistance, making successful treatment and control of the disease even more difficult. The third report on global surveillance for tuberculosis drug-resistance reveals alarming levels of MDR-TB (resistance at least to isoniazid and rifampicin) of up to 14% among new cases, with an estimated 300,000 new cases of MDR-TB globally per year [3]. Of particular concern are parts of Eastern Europe and Central Asia where tuberculosis patients are 10 times more likely to have MDR-TB than in the rest of the world [4].

The treatment of patients with MDR-TB is extremely difficult, expensive, and requires special treatment regimens and case management [5]. In addition, patients infected with MDR-TB may remain infectious for prolonged periods of times further accelerating the spread of MDR-TB. It is therefore important to understand factors contributing to the development of MDR-TB and the potential epidemiological impact of MDR-TB strains in order to develop effective control strategies.

Increasing tuberculosis incidence and the emergence of MDR-TB in the former Soviet Union have been mainly attributed to the deterioration of economic and social conditions as well as to the widespread breakdown of tuberculosis control systems since the late 1980s [6,7]. The possibility that the pathogen itself is also contributing to this problem has been recently suggested by two studies from the Russian Federation, which found high proportions of a particular genotype of tuberculosis, namely the Beijing genotype, which was strongly associated with drug resistance [8,9].

This notion has been further supported by recent studies that have provided evidence that the genetic heterogeneity of *Mycobacterium tuberculosis *complex isolates is greater than previously thought, and might influence the transmissibility and virulence of particular isolates [10-13]. Strains of the Beijing genotype were first described in China and neighbouring countries in 1995 [14], and subsequently the occurrence of Beijing genotype strains has been documented in several parts of the world [8,9,14-17]. The Beijing genotype has caused outbreaks of MDR-TB [18,19], and some, but not all studies, indicate an association with drug resistance [16]. However, there is limited information available on both the occurrence and effects of the Beijing genotype, especially from areas with high tuberculosis incidence and high rates of MDR-TB. If these strains have an enhanced capacity to gain resistance, this will have serious consequences for the treatment of tuberculosis.

In a recent study, we found high levels of MDR-TB in the Aral Sea region in Uzbekistan and Turkmenistan, Central Asia [20]. A cross-sectional survey of more than 400 smear-positive tuberculosis patients, revealed levels of MDR-TB of 27% in Karakalpakstan (Uzbekistan) and 11% in Dashoguz (Turkmenistan). The DOTS strategy was introduced progressively into this region from 1998 and now covers a population of around 4 million people. High case notification rates for smear positive tuberculosis: 190/100,000/year in Karakalpakstan and 70/100,000/year in Dashoguz are reported from the DOTS programme.

In this study, we elucidate the importance of the Beijing genotype for tuberculosis in the Aral Sea region. Molecular typing of the isolates from the drug resistance survey was performed to determine the proportion of patients infected with Beijing genotype strains, and associations with drug resistance and other patient characteristics were analysed.

## Materials and methods

### Study population

A cross-sectional survey for anti-tuberculosis drug-resistance was conducted in 4 districts in the Autonomous Republic of Karakalpakstan, Uzbekistan, and in 4 districts in Dashoguz Velayat, Turkmenistan. Smear positive pulmonary tuberculosis patients initiating DOTS treatment in these districts were included in the study. The study was based on the recommendations for drug resistance surveys outlined by WHO and IUATLD [21]. A description of the study design, patient recruitment and data collection can be found in an earlier paper [20]. Written informed consent was obtained from all patients.

### Primary isolation and drug susceptibility testing

Sputum specimens were shipped from Uzbekistan and Turkmenistan throughout the survey to the Supra-National Reference Laboratory (SRL) in Borstel, Germany. Primary isolation of mycobacterial isolates was performed as described elsewhere [22]. All isolates were identified as *M. tuberculosis *using gene probes (ACCUProbe, GenProbe, San Diego, USA), and standard biochemical procedures. Drug susceptibility testing (DST) was performed by using the proportion method on Löwenstein-Jensen medium and/or the modified proportion method in BACTEC 460TB (Becton Dickinson Microbiology Systems, Cockeysville, USA) according to the given instructions.

### IS*6110 *DNA RFLP fingerprinting and spoligotyping analysis

Extraction of genomic DNA from the mycobacterial strains and DNA fingerprinting using IS*6110 *as a probe were performed according to a standardized protocol as described elsewhere [23]. Additionally, all isolates were analysed by the spoligotyping technique as described previously by Kamerbeek et al. [24]. The molecular typing data were analysed with the Bionumerics software (Windows NT, version 3.0; Applied Maths, Kortrijk, Belgium) as instructed by the manufacturer. The spoligotyping data were used to additionally confirm strain relationships and for the identification of Beijing genotype isolates (no hybridization to spacers 1–34, hybridization to spacers 35–43).

### Statistical analyses

All clinical and laboratory data were entered into a database using Epi-info (6.04, CDC. Atlanta, GA, USA). Chi square analysis was used for comparisons of proportions. Logistic regression analysis was performed to identify variables independently associated with MDR-TB and Beijing genotype (SPSS version 10.0, SPSS Inc., Chicago, IL, USA).

## Results

Out of the 416 strains included in the drug resistance survey, 397 were available for the IS*6110 *DNA fingerprint and spoligotyping analysis performed in this study (19 strains did not grow at the time the DNA fingerprinting was started). This subset is comprised of 208 patients from Karakalpakstan and 189 patients from Dashoguz. The characteristics of the patients included in this molecular investigation did not differ from the characteristics of the complete study population of the drug resistance survey (data not shown). In brief, the final sample consisted of 239 male (60%) and 158 female (40%) patients. The age of the patients ranged from 11 years to 77 years, with a mean of 34 years. Across both regions, 203 were new cases (51%) and 194 (49%) had received previous tuberculosis treatment.

### Molecular typing results

**Figure 1:**
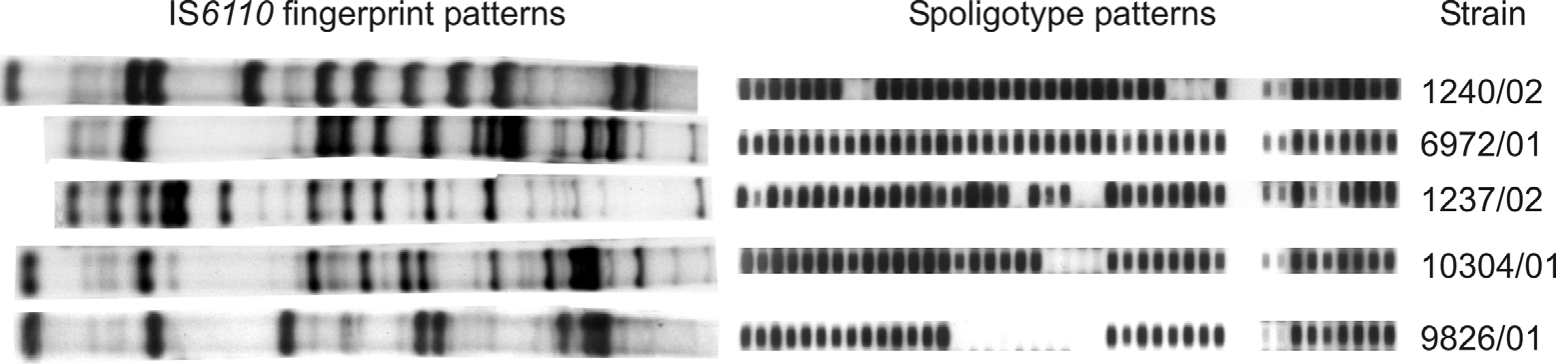
IS*6110 *DNA fingerprint and spoligotype patterns of the isolates obtained from five randomly chosen patients with double infections.

In general, a high degree of diversity of IS*6110 *DNA fingerprint patterns was observed among the strains analysed. For 382 isolates, clear-cut IS*6110 *banding patterns were obtained, while, 15 strains (3.8%) showed mixed banding patterns demonstrating double infection with two *M. tuberculosis *strains (Fig. 1). These findings could be confirmed by the presence of mixed spoligotype patterns showing hybridization to the Beijing-typical spacers 35 to 43 and to spacer sequences not present in Beijing genotype strains (Fig. 1). In all cases of double infection identified, the patients were infected with a non-Beijing and a Beijing genotype strain, mainly with a weak Beijing genotype background pattern (Fig 1). All typing experiments were repeated for these strains to exclude the possibility of DNA carry over contamination. Since no clear IS*6110 *band definition is possible in mixed strain isolates, the patients with mixed infections were excluded from further investigations. Resistance to first-line drugs among the remaining 382 isolates, stratified by previous tuberculosis treatment and Beijing genotype, are shown in Table 1.

**Table 1:** Resistance to anti-tuberculosis drugs stratified by previous tuberculosis treatment and for patients infected with a Beijing or a non-Beijing strain.

	**New cases**	**Previously treated**	**Total**	**Beijing**	**Non-Beijing**	**OR (Beijing) (95% CI)**
Total cases	198	184	382	190	192	
Resistance to:						
Ethambutol	14 (7%)	47 (26%)	61 (16%)	49 (26%)	12 (6%)	5.2 (2.6–10.8)
Rifampicin	15 (8%)	54 (30%)	69 (18%)	51 (27%)	18 (9%)	3.6 (1.9–6.6)
Pyrazinamide	6 (3%)	24 (13%)	30 (8%)	22 (12%)	8 (4%)	3.0 (1.2–7.6)
Streptomycin	68 (3%)	111 (60%)	179 (47%)	114 (60%)	65 (34%)	2.9 (1.9–4.6)
Isoniazid	47 (24%)	108 (59%)	155 (41%)	99 (52%)	56 (29%)	2.6 (1.7–4.1)
MDR-TB	15 (8%)	53 (29%)	68 (18%)	51 (27%)	17 (9%)	3.8 (2.0–7.1)

**Figure 2:**
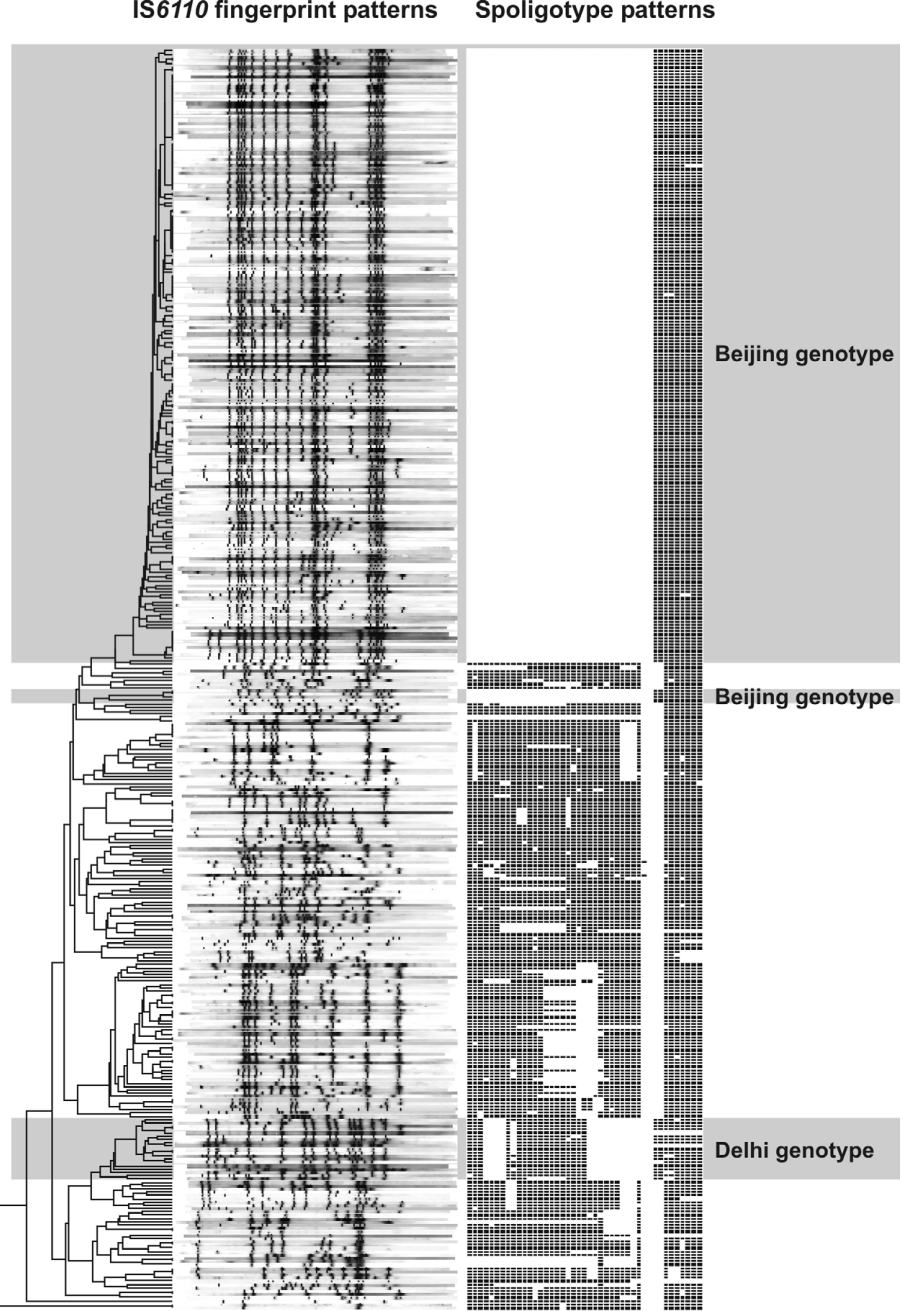
IS*6110 *DNA fingerprint and spoligotype patterns of the 382 strains analysed. Banding patterns are ordered by similarity in a dendrogram.

To determine prominent genotypes and strains with identical IS*6110 *and spoligotype patterns, a dendrogram was calculated based on the similarity of the IS*6110 *RFLP patterns (Fig. 2). Among the 382 strains included in this analysis, 190 (49.8%) were of the Beijing genotype (Fig. 2). The majority of Beijing genotype isolates showed the typical spoligotype pattern (hybridization to all of spacers 35–43 and no hybridization to spacers 1–34) and "classical" Beijing genotype IS*6110 *RFLP patterns with a similarity of more than 70% (Fig. 2). Thus, these isolates formed a well-defined branch in the dendrogram. However, three isolates had further spacer deletions and did not hybridize to spacers 37 and 38, 41, 42 or 43, and 40 and 41, respectively. Furthermore, another four strains showed the typical Beijing genotype spoligotype pattern, but had IS*6110 *patterns not showing the characteristic Beijing genotype signature (Fig. 2).

Surprisingly, we also identified a number of isolates (n = 19, 5%) that belonged to the Delhi genotype, which has been found to be the dominant strain type in the Delhi region of India [25]. These strains showed highly similar IS*6110 *RFLP patterns thus also forming a certain branch in the dendrogram and had spoligotype patterns described to be typical for this genotype.

Based on identical RFLP and spoligotype patterns, 152 strains (40%) were grouped in 42 clusters ranging in size from two to 21 cluster members as follows: 26 clusters with 2 isolates, 4 with 3 isolates, 4 with 4 isolates, 3 with 5 isolates, 2 with 7 isolates, 1 with 8 isolates, 1 with 14 isolates, and 1 with 21 isolates. Although a third of these strains were in small clusters with just 2 isolates (n = 52 isolates), a remarkable number of strains were in the 2 largest clusters (n = 35), together representing 23% of all clustered isolates and 9% of all strains included in the cluster analysis. All larger clusters (n ≥ 4) were composed of Beijing genotype strains and, overall, 104 (68%) out of the 152 clustered isolates belong to the Beijing genotype. Overall, 55% of Beijing strains were clustered compared to 25% of non-Beijing strains (p < 0.01). There was no statistically significant difference in clustering between new and previously treated cases, between sexes or among age groups.

### Characteristics associated with Beijing genotype strains

To define any association of drug resistance with Beijing genotype, we determined the percentage of Beijing genotype strains among different categories of drug resistance and by previous tuberculosis treatment status (Table 2). The association between Beijing genotype and levels of drug resistance was strikingly similar for both new and previously treated patients, with increasing proportions of Beijing type observed among categories of drug resistance (chi squared, p ≤ 0.001). While only 38% of the fully susceptible isolates belonged to the Beijing genotype, 75% of MDR-TB patients were infected with Beijing strains. The association between Beijing genotype and individual drug resistance, although evident for all first-line drugs, was not consistent (Table 1). There was a stronger association with ethambutol resistance, followed by rifampicin and pyrazinamide resistance, with MDR-TB closely mirroring the association with rifampicin resistance (Table 1). In contrast to the Beijing type, the smaller number of strains identified to be of the Delhi genotype was not associated with drug resistance when compared to strains not of the Beijing or Delhi families.

**Table 2:** Percentage of Beijing genotype isolates among different categories of drug resistance, by tuberculosis treatment status.

		**Total cases**	**Beijing infection (%)**
**New cases**	Fully susceptible	124	48 (39%)
	Resistant to one drug only	35	16 (46%)
	Poly-resistant (not MDR-TB)	24	14 (58%)
	MDR-TB	15	11 (73%)
	Total	198	89 (45%)
**Previously treated cases**	Fully susceptible	53	20 (38%)
	Resistant to one drug only	37	17 (46%)
	Poly-resistant (not MDR-TB)	41	24 (59%)
	MDR-TB	53	40 (76%)
	Total	184	101 (55%)

Previously, we have reported a logistic regression model identifying factors related to MDR-TB infection in this region [20]. Significant factors were previous tuberculosis treatment, residence in Karakalpakstan and female gender. When Beijing genotype is added to this model, it joins these factors as a significant independent predictor of MDR-TB (OR = 3.6 95%CI 1.9–6.8).

To investigate patient factors associated with Beijing genotype, univariate and multivariable analyses were performed with Beijing genotype infection as the dependent variable. Factors analysed were: previous tuberculosis treatment, belonging to a cluster, region (Karakalpakstan or Dashoguz), sex, previous imprisonment, reported contact with a tuberculosis case, alcohol use, accompanying illness, and age. Within univariate analysis, the only significant association observed was with clustering (OR = 3.6, Table 3). This result was confirmed in the multivariable analysis.

**Table 3:** Factors associated with Beijing genotype infection (univariate and multivariable analyses).

	**No.**	**Beijing**	**Univariate OR (95% CI)**	**Multivariable OR (95% CI)**
Previous TB treatment	184	101	1.5 (1.0–2.2)	1.3 (0.8–2.0)
Being in a cluster	152	104	3.6 (2.4–5.6)	3.5 (2.3–5.5)
Region (Karakalpakstan)	198	107	1.4 (1.0–2.2)	1.3 (0.8–1.9)
Female gender	156	75	0.9 (0.6–1.3)	1.1 (0.7–1.8)
Previous imprisonment	67	40	1.6 (1.0–2.8)	1.3 (0.7–2.5)
Close contact with a TB case	39	21	1.2 (0.6–2.3)	1.0 (0.5–2.0)
Alcohol use	30	20	2.1 (1.0–4.7)	2.0 (0.8–4.6)
Accompanying illness	48	19	0.6 (0.3–1.2)	0.6 (0.3–1.3)
Age over 30	202	102	1.0 (0.7–1.6)	1.1 (0.7–1.8)
**Total**	**382**	**190**		

## Discussion

This study demonstrates that the Beijing genotype is a major cause of tuberculosis in this high incidence region of Central Asia. A strong association with drug resistance has been documented, independent from previous tuberculosis treatment. In addition, an association of Beijing genotype with clustering suggests that these strains are being transmitted throughout the community, possibly mediating the transmission of drug-resistant tuberculosis in this setting.

Since its description in 1995, the Beijing genotype of *M. tuberculosis *has elicited increasing attention. The prevalence of the Beijing genotype shows strong geographical variation from below 10% to more than 90% of the strains analysed [16]. High rates of Beijing genotype strains have been reported from some regions in Eastern Europe, such as Estonia, Azerbaijan, and Russia (Samara oblast, Archangel oblast, north-western region), while in Western European countries the prevalence of Beijing genotype is low [8,9,16,26-32]. Although a high prevalence of Beijing genotype strains appears to be confirmed for some regions of the world, direct comparison of data is difficult due to variations in strain identification methodology and different survey inclusion criteria utilised. Many of the studies performed so far have addressed subpopulations such as prison inmates or have provided only limited information on inclusion criteria [28,29,32]. These studies indicate high rates of Beijing genotype strains among these sub-populations, but definitive conclusions regarding prevalence in the general population are more difficult.

This study presents results on the prevalence of Beijing genotype among a representative cross-sectional sample in Central Asia. Since 50% of patients were found to be infected with Beijing genotype isolates, the data obtained confirm the importance of the Beijing genotype in this region and place this region in Central Asia among the Beijing genotype "high incidence" regions. Infection with Beijing genotype was not associated with particular subgroups of patients such as previous prison inmates or with a specific region suggesting that tuberculosis due to Beijing genotype is not restricted to specific parts of the population, but affects the general community.

This is of importance, since the Beijing genotype was additionally found to be significantly associated with drug-resistance and might be a driving force for the spread and emergence of MDR-TB. It is well known, that strains of the Beijing genotype have been involved in outbreaks of drug-resistant tuberculosis such as in the USA and in Russia [18,19]. However, there was no consistent association with drug resistance among 12 studies evaluated in the review of Glynn and colleagues [16]. Only three studies found a strong association with resistance to particular drugs, while the other studies showed none or even a negative association.

In this investigation a strong association between the Beijing genotype and resistance to single drugs as well as with MDR-TB was observed. Beijing genotype was also associated with clustering when compared to non-Beijing strains, indicative of more recent transmission [33]. These data are in accordance with the results of the few recent studies so far performed in Eastern Europe: addressing tuberculosis in prison populations [28,32], and the general population in the Archangel and Samara Oblasts and north-western regions of Russia and Estonia [8,27,29]. It is therefore likely that the Beijing genotype is a major cause of tuberculosis and particularly MDR-TB in wide areas of the former Soviet Union.

From our data, we cannot tell whether the Beijing genotype has emerged recently in the Aral Sea region or whether its long-term presence has been revealed. However, data from the Archangel oblast indicate that the Beijing genotype has been introduced in this region recently and has been strongly disseminating during the last few years [8]. Additionally, the Beijing genotype has also been reported to infect a rising number of patients on Gran Canaria Island, Spain, adding to the conclusion that strains of the Beijing genotype might have a selective advantage compared to other *M. tuberculosis *strains and spread more rapidly [34]. Therefore, in the former Soviet Union, the introduction of Beijing strains may have coincided with the deterioration of socio-economic conditions and the tuberculosis control system, and together this combination may contribute to the current MDR-TB epidemic.

The presence of multiple infection in 4% of the samples in this study confirms previous findings reporting mixed infections among small numbers of patients [35,36]. The simultaneous infection of patients with a non-Beijing and a Beijing strain determined here, might point to specific properties of the Beijing genotype. In the majority of cases identified, the fingerprint pattern typical for the Beijing genotype was weak compared to the other pattern, indicating that an exogenous re-infection with a Beijing genotype strain might have occurred in these patients, which then out-competed the first isolate. A high rate of patients simultaneously infected with a Beijing and non-Beijing strain was also reported in a recent study in Cape Town, South Africa [37]. In this investigation, 19% of all patients were simultaneously infected with Beijing and non-Beijing strains. The possibility that resistant Beijing genotype strains can efficiently super-infect patients receiving regular tuberculosis treatment was further suggested by cases of exogenous re-infection with MDR-TB Beijing isolates described in previous studies [38,39]. These findings suggest that susceptible or resistant Beijing genotype strains can potentially re-infect tuberculosis patients and in the case of resistant strains even whilst they are receiving regular tuberculosis therapy. This would be expected to result in a selective advantage for Beijing strains and would lead to a higher Beijing prevalence among drug resistance isolates.

Future studies are needed to clarify the characteristics of this important genotype of *M. tuberculosis*. Possible variations in host-pathogen interactions among strains of various genotypes needs to be identified and the role of Beijing genotype infection as a possible risk factor for treatment failure and/or drug resistance development must urgently be addressed in longitudinal studies in the affected high incidence regions. In short, the effect of this genotype on tuberculosis control efforts need to be further investigated.

## Competing interests

The author(s) declare that they have no competing interests.

## Authors' contributions

Helen Cox: Conception and design of the study, acquisition, analysis and interpretation of data, drafting and revising of the article, given final approval to this version to be published

Tanja Kubica: analysis and interpretation of data, drafting and revising of the article, given final approval to this version to be published

Daribay Doshetov: analysis and interpretation of data, drafting and revising of the article, given final approval to this version to be published

Yared Kebede: Conception and design of the study, drafting and revising of the article, given final approval to this version to be published

Sabine Rüsch-Gerdes: Conception and design of the study, interpretation of data, drafting and revising of the article, given final approval to this version to be published

Stefan Niemann: Conception and design of the study, acquisition, analysis and interpretation of data, drafting and revising of the article, given final approval to this version to be published
